# Canadian Metadata Catalogue for Health Science Research

**DOI:** 10.23889/ijpds.v11i1.3372

**Published:** 2026-07-09

**Authors:** Allan Garland, Peter Dodek, Kednapa Thavorn, Rita Wissa, Wendy Sligl, Wynona Marleau, M. Elizabeth Wilcox, Isabel Fortier, for the Sepsis Canada Network

**Affiliations:** 1 Department of Medicine, University of Manitoba, Winnipeg, Manitoba, Canada R3A 1R9; 2 Department of Medicine, University of British Columbia, Vancouver, British Columbia, Canada V5Z 1M9; 3 Ottawa Hospital Research Institute, The Ottawa Hospital and School of Epidemiology and Public Health, University of Ottawa, Ottawa, Ontario, Canada K1H 8L6; 4 Maelstrom Research, McGill University, Montreal, Quebec, Canada H3G 1A4; 5 Department of Critical Care Medicine, University of Alberta, Edmonton, Alberta, Canada T6G 2B7; 6 Sepsis Canada Network, Hamilton, Ontario, Canada L8L 2X2; 7 Research Institute of the McGill University Health Center, McGill University, Montreal, Quebec, Canada H3A 2R7

**Keywords:** metadata catalogue, Canada, health science research, medical data, social determinants of health

## Abstract

**Introduction:**

While Canada is rich in databases useful to support healthcare research, they are widely distributed, often poorly documented, and it is challenging to identify relevant databases, apply for access, and eventually use, link or harmonise the data. Even if the databases needed to address specific questions are known, it is difficult and time-consuming to find the metadata, the “data about the data” required to understand the characteristics and data content of these resources. A solution to these challenges is creation of metadata catalogues, which detail metadata for multiple databases, not the actual data.

**Objectives:**

Describe a new catalogue including metadata about Canadian medical and non-medical databases’ characteristics and variables, and information to assist catalogue users in seeking data access.

**Methods:**

Starting with a list of 385 national, provincial and regional databases, a group of physician-investigators, epidemiologists, data scientists and patient partners prioritised databases for inclusion. Metadata cataloguing occurred in steps: (i) description of the database with listing of its characteristics, and when available, (ii) addition of information about collected variables.

**Results:**

83 individual databases are documented in the Metadata Catalogue of the Sepsis Canada Network (https://www.maelstrom-research.org/network/sepsis). 57 are registries, 13 are cohort and 13 cross-sectional databases. 16 cover all of Canada, while another 13 cover most of the country; 45 focus on a single province. For 33 databases (38%) the catalogue includes detailed information about variables collected.

**Conclusions:**

This metadata catalogue includes databases collecting information spanning the continuum of medical care, non-medical data, and determinants of health. It is freely available online and extensively searchable. It can facilitate implementation of a wide range of research initiatives into medical conditions, medical care, and outcomes.

## Introduction

Although Canada is rich in national, provincial and regional population-based databases, they are widely distributed, and there are challenges in identifying relevant databases, applying for access, linking and harmonising the data [1]. Many such databases are not well known to researchers and medical experts seeking to answer research questions. Individual-level data of interest for these investigators includes health data, causes of death, lifestyle and behaviours, socioeconomic status, and others. Area-level data is also of interest and includes social determinants of health (e.g. the national Census), and the built environment (e.g. the Canadian Urban Environmental database) [2–6].

The full potential of existing data required to answer epidemiologic questions derives from using deterministic or probabilistic methods [7–9] to co-analyse different types of data [10, 11]. For example, assessing the impact of diet on the incidence of a health disorder requires co-analysing data identifying individuals by disorder status, with their dietary information. Even if the databases collecting such information are known, it is challenging for external users to find the information required to ask for access to, and use the data: host institution, number of participants, rules for data access, data collection timepoints, data sources, list of variables with definitions and format, missing data indicators, persistent identifiers included, and others. These metadata, or “data about the data”, are necessary to determine if a database is appropriate for achieving investigators’ goals. Many data-related challenges to research in Canada are being addressed by the Health Data Research Network, [1, 12] but documenting metadata is not part of its work (K. McGrail, personal communication).

While there is a common set of organisational elements for Canadian federal data sources, [13] no national metadata standards exists for documenting information and coding variables. For example, in diagnosis coding within provincial/territorial outpatient medical claims data, some use ICD-9 coding while others use ICD-10, some allow only one diagnosis, others multiple, and some limit to three digit diagnosis codes while others use five digits [1]. It is thus essential to provide comprehensive information to external users interested in using the collected data. However, comprehensive metadata is not readily available for all existing databases and even databases held by a single data custodian sometimes record metadata differently. One approach to coping with these challenges is creating metadata catalogues. Metadata catalogues document information for multiple databases, not the actual data [14, 15]. By reporting information about the databases and how variables are collected and coded, a metadata catalogue facilitates identification of databases of interest to answer specific research questions and exploration of the potential to harmonise or link selected databases. For maximal usefulness, metadata catalogues should allow querying for data items based on desired attributes. These catalogues are rare in healthcare, with most containing registries or observational cohort studies related to single diseases, and representing small catchment areas [11, 15].

Here we describe the new, Sepsis Canada Network catalogue including metadata about Canadian medical databases enabling identification of individuals with clinical disorders, non-medical databases containing diverse information about Canadians, and database designs, variables collections and information to assist catalogue users in seeking data access. Although creation of this metadata catalogue was motivated by participation in the Sepsis Canada Network, a Canadian Institutes of Health Research-funded organisation creating national infrastructure to facilitate research on sepsis, [16] it is not specific to sepsis, and can be used to investigate a wide variety of health disorders.

## Methods

Database criteria setting and selection were done from September 2021 to February 2024, by a group comprising six physician-investigators, two epidemiologists, two data scientists with metadata expertise, and one partner with lived sepsis experience. We contracted with Maelstrom Research (https://www.maelstrom-research.org/), a research project of the Research Institute of the McGill University Health Centre, to include the Sepsis Canada Network in the Maelstrom catalogue, using their listing standards, and to host it on the internet [15, 17]. Appendix 1 contains additional details of the Maelstrom web interface. Contributing to the Maelstrom catalogue enabled inclusion of a specific group of databases, while also allowing users to have access to the information contributed by 30 additional national and international networks. The catalogue is extensively searchable, including by: data source and type, data field categories, data fields, jurisdiction, years included, number of records, and others. This report only concerns the Sepsis Canada Network documented on the Maelstrom Research catalogue. Initial criteria for database inclusion were: >10,000 subjects, except for clinical databases for which the minimum would be 1000; availability of data for use by others than the original investigator/custodian; and could be of interest to link to other databases.

We began with a list of potential databases comprising the 385 listed as of January 2021 by the Health Data Research Network (HDRN Canada) [12], augmented by others known to the investigator group. We included individual-level and small area-level databases [4]. We did not include data from interventional sepsis treatment trials, as they generally are small, quickly become dated as they cover a restricted time interval, and are only linkable to other databases if the informed consent included future linkage, which virtually no trials have done as of yet. These were organised into a spreadsheet listing: region(s), host institution, design, whether or not participant selection was population-based, number of participants, years covered, whether or not information was self-reported, whether or not data was derived from clinical records, and key measures collected.

We segmented the process of selecting databases (Table 1). For each segment: (i) we chose a focus or foci, (ii) identified databases for potential inclusion based on ‘i’, (iii) each member of the investigative team prioritised the databases from ‘ii’, (iv) collated the individual priorities, and (v) selected databases to include by consensus, based on ‘iv’. The number of databases included was limited by the funding and duration envelopes for this project. After obtaining permission, selected databases already included in the Maelstrom catalogue were attached to the Sepsis network.

**Table 1:** Database Selection Segmentation Details

**Segment**	**Focus/foci**
1	Most valuable national databases; regional intensive care unit clinical databases
2	Additional national databases; Most valuable provincial/regional databases
3^*^	Information on: diabetes mellitus, diet, alcohol use, immune-mediated disorders, immigration, income
4	Suggested after email solicitation of all members of the Population Health pillar of the Sepsis Canada Network
5	Additional high-priority provincial/regional databases

^*^list of potential databases augmented by consultation with Canadian content experts outside the main investigator group.

For each selected database we sought its metadata online, and by direct contact with the principal investigator or data custodian. We eliminated those for which we were unable to obtain sufficient information or permission for inclusion. Metadata cataloguing occurred in two steps: (i) description of the database characteristics and when available, (ii) addition of information about collected variable.

After inclusion in the catalogue, database-specific metadata is updated or corrected if the cataloguing team find errors during regular checks, or if principal investigators or custodians requests modifications. Maelstrom was launched in 2011 and aims to pursue long-term maintenance of the catalogue website, including the Sepsis network.

## Results

The Sepsis Canada Network catalogue currently includes 83 individual databases (eTable 2) and documents: design, participant profiles, timing of data collection(s), and contact information to seek access to data. Fifty-seven databases are registries, 13 are cohorts and 13 are cross-sectional databases. Sixteen databases cover all Canadian provinces and territories, and another 13 cover most of the country; forty-four focus on a single province. Twelve databases collected biological samples, variably comprising blood, urine, saliva and nail samples. Sixty-three databases (75%) are still accumulating records. For 33 databases (38%) the metadata catalogue includes detailed information about collected variables. In addition to the 83 databases, our metadata catalogue contains a link (eFigure 5) to the Canadian Urban Environmental Health Research Consortium (CANUE), which contains small area-level data on four environmental domains: greenness, neighbourhood, air quality, and weather [6].

Centralised medical databases that cover all or most of Canada include inpatient care (eTable 2 item 17), outpatient and emergency department care (item 32), cancer diagnoses and treatment (item 1), chronic care (item 28), home care (item 31), rehabilitation care (item 29), and births and deaths (items 4, 5). For many of these, contact information is provided for their provincially available versions (eFigure 7). Other medical databases include population-based participants from one or more provinces regarding: prescription drug dispensation (eTable 2 items 26, 50, 52, 70, 72, 75), diagnostic laboratory test results (items 55, 61 68, 78), trauma (items 15, 62, 65), burns (item 38), Multiple Sclerosis (items 45, 58), patients admitted to intensive care units (items 43, 57, 73), and immunisation (item 56).

National non-medical databases include immigration data (eTable 2, item 11) and income taxes (items 10, 16). Our metadata catalogue also covers metadata from a number of national, population surveys (items 2, 3, 8, 18, 19, 22-24, 25). The national Census of Population, conducted every five years, is also included (item 6) [18]. The Census contains information [4] on age, sex, education, household structure, ethnicity, immigration, language, labor force status, household income, and the domains of the Canadian Index of Multiple Deprivation [19]. Furthermore, the long-form Census, sent to 25% of the households, contains individual-level information on those parameters, and additionally on activities of daily living, religion, household expenditures, military service, and First Nations, Metis and Inuk status [20].

**Figure 1:**
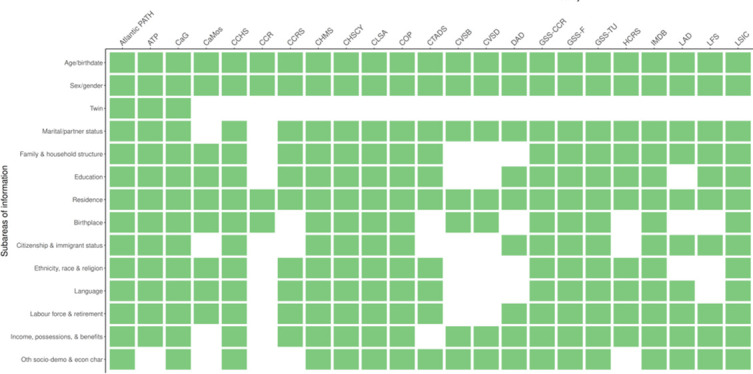
Illustration of Cross-Referencing of Sub-Domains Within the Socio-Demographic and Economic Domain (indicated along the left side) for 23 of the 33 datasets with detailed variable information (indicated across the top)

The Maelstrom catalogue website is powered by the OBiBa MICA software [21, 22] developed by Epigeny (France). The catalogue has extensive search and filtering capabilities, for which a tutorial is available at https://www.maelstrom-research.org/page/tutorials?topic=search. To facilitate searchability and data exploration, variables are classified under 18 major domains (Table 2) and 134 sub-domains (eTable 1). For the databases with detailed variable information, search and cross-referencing graphics are easily generated on the Maelstrom website, showing available sub-domains (Figure 1, eFigures 11A, 11B).

**Table 2:** Distribution of 18 Major Areas of Information Contained in the Sepsis Canada Network Metadata Catalogue

**Area**	**# databases**	**Area**	**# databases**
Sociodemographics and economic characteristics	78	Death	19
Lifestyle and behaviours	29	Physical measures and assessments	26
Birth, pregnancy, reproductive health history	13	Laboratory measures	26
Perceptions of health, quality of life, developmental and functional limitations	29	Cognitive, personality and psychological measures & assessments	25
Diseases	54	Life events, life plans, beliefs & values	21
Symptoms and signs	19	Preschool, school and work life	19
Medications and supplements	37	Social and environmental relationships	26
Non-pharmacologic interventions	39	Physical environment	24
Health and Community Service Utilisation	52	Administrative information	78

Persistent identifiers for deterministically linking databases (e.g. personal health identifier number, social insurance number, hospital record number), when provided, are categorised within Maelstrom under the subcategory “Identifiers” of the category “Administrative Information”. The Variables Content Summary at the bottom each database’s page has a link to included identifier variables (eFigure 12); clicking on it opens this list (eFigure 13).

## Discussion

To address the need from the scientific community to optimise use of existing data sources, we created a metadata catalogue that can assist investigators in identifying databases of interest and persons to contact to access Canadian databases containing information relevant to a wide range of research questions on sepsis and many other health conditions. The catalogue is freely available online and searchable.

Identifying health disorders is a vital part of epidemiological research. Our metadata catalogue includes administrative databases spanning the continuum of medical care and allows searching for information collected about many acute [23–26] and chronic [27, 28] disorders. It also includes surveys, e.g. the Canadian Community Health Survey (CCHS), a cross-sectional survey of Canadians which randomly samples 130,000 adults every other year, contains self-report information about the presence of 39 disorders [29]. Assessment of self-report indicates that it is adequately accurate for some, but not all disorders [30, 31].

The catalogue also helps investigators identify non-medical parameters they are interested in exploring, such as lifestyle and social determinants of health [32, 33]. Ongoing, national-level, population surveys collecting non-medical information are documented in the catalogue and include the Canadian Community Health Survey [29], Canadian Health Survey on Children and Youth [34], Canadian Longitudinal Study on Aging [35], Canadian Health Measures Survey, [36] General Social Survey [37], and Labour Force Survey [38]. A salient example is the CCHS, which asks about sexual orientation, household living arrangement, marital status, education, immigration, activities, function, health, mental health, use of health services, diet, food insecurity, life satisfaction, life stress, work, and use of cigarettes, alcohol, cannabis and illicit drugs [39].

Although the primary goal of our note catalogue is to delineate the existence and contents of Canadian databases useful in epidemiologic research, its value is amplified by the possibility to explore harmonisation potential across databases and interest for linkage. However, the substantial effort required to obtain the actual data and perform linkage is beyond the scope of the catalogue, or this manuscript. While the identifiers included in the databases are documented in the catalogue, the challenges to be faced when accessing or linking data (limited access to data, specific conditions to respect, capacity to transfer data to an external user, presence of compatible identifiers, and others) need to be solved by investigators when requesting note modification access to data. HDRN Canada provides access to tools and resources to assist investigators with data linkage in the Canadian context. The HDRN Data Access Support Hub (DASH) can assist with linkage within a single jurisdiction, and its DASH Team is available to provide specialised guidance for linking data across jurisdictions [40]. HDRN also offers coordination services to help researchers link administrative health records with area-level social determinants of health.

While valuable, the Sepsis Canada Network catalogue has limitations. First, although we prioritised inclusion of the most useful databases for epidemiological research, other valuable Canadian databases could not be included due exhaustion of project funding. This leads to some limitations on the scope of research questions it can help to explore. For example, although it includes Canadian hospitalisations, day surgeries, inpatient rehabilitation, homecare, and long-term care, its coverage for outpatient and emergency department care are not universal. Second, for some databases we were unable to obtain the list of all variables collected, leading to a comprehensive, but occasionally incomplete list of variables. However, the information included enables investigators to identify databases of interest and seek further detailed information from data custodians. Third, for some databases we were unable to obtain data field details. However, the included descriptions and contact information enables investigators to make preliminary assessments and seek further detailed information, which is generally available from data custodians for those seeking data access. Finally, the catalogue documents 83 Canadian databases as they existed in 2024. If requested by principal investigators/custodians, information content is updated, and we hope the catalogue will retain value over time. However, changes will occur that will not be covered by these requests. Many of the contributing databases have operated for decades and are relatively stable, even if specific data collection events or variables are added or personnel structure and governance evolve. In the long-term, even if information is increasingly missing or outdated, we expect the catalogue will remain useful by mapping the existence and scope of key Canadian, health-relevant data holdings, clarifying where and how information can be found. In order to do so, the Maelstrom team regularly seeks funding to improve the catalogue content and structure, update information content, add new databases and include new variables collected by ongoing databases. Specifically for the Sepsis Canada catalogue, we will seek funding to expand and regularly update the metadata catalogue, beginning in 2029, after it has been in existence for five years.

## Conclusions

This work describes a new, Canadian metadata catalogue aiming to enhance the discoverability and use of Canadian health and social data. The catalogue supports multidisciplinary research by streamlining knowledge about medical and non-medical databases. The catalogue will need to be maintained but also evolve through time. Future research should evaluate its impact, explore potential for expansion, and investigate opportunities to optimise metadata collections using rapidly evolving technological capacities.

## Data Availability

This metadata catalogue is available at: https://www.maelstrom-research.org/network/sepsis.
